# A randomized clinical study to assess the performance of a marketed denture adhesive in a model of food infiltration in healthy, edentulous adults

**DOI:** 10.1002/cre2.703

**Published:** 2022-12-13

**Authors:** Nisha Patel, Roshan Varghese, Gary R. Burnett, Mounir Atassi, Kimberly Milleman, Jeffery Milleman

**Affiliations:** ^1^ GSK Brentford Middlesex UK; ^2^ Haleon Weybridge Surrey UK; ^3^ Salus Research Inc. Fort Wayne Indiana USA

**Keywords:** adhesives, dentures, denture retention, methods

## Abstract

**Objectives:**

An optimized food infiltration methodology was utilized to assess the objective and subjective efficacy of a marketed denture adhesive regarding denture dislodgment and infiltration and perception of food particles under maxillary and mandibular dentures. A pilot study helped optimize methodologies before the efficacy study.

**Materials and Methods:**

Participants were healthy adults (*n* =48 for both studies) with fair‐ to well‐fitting and well‐made full maxillary and mandibular dentures. In the pilot, groups were a denture adhesive applied in a conventional dabbed‐on pattern, a denture adhesive applied in continuous strips, or no adhesive. In the efficacy study, groups were the Test denture adhesive (continuous strips pattern application) or no adhesive, employed in a crossover design. Food infiltration was investigated through measurement of peanut particle mass retrieved from under each denture (30–32 g chewed). No formal statistical testing was performed in the pilot. Statistical analysis in the efficacy study was performed using analysis of variance. Primary efficacy evaluation was combined peanut particle mass from both dentures. Secondary efficacy evaluations included peanut particle mass under separate dentures, participant‐reported denture dislodgements, and awareness/rates of how bothersome peanut particles under dentures were.

**Results:**

In the pilot, the median peanut particle mass was lower with either pattern application compared with no adhesive. In the efficacy study, peanut particle mass under combined dentures was lower with than without adhesive (geometric mean [product of values]: 5.56 vs. 29.13 mg) with a between‐group geometric mean ratio (adhesive over no adhesive) of 0.19 (95% confidence interval: 0.12, 0.30) favoring the Test adhesive (*p* < .0001). Similar Test adhesive beneficial outcomes in both studies included significantly fewer denture dislodgements and awareness and how bothersome peanut particles under dentures were. Treatments were generally well‐tolerated.

**Conclusions:**

These findings, including reduced peanut particle infiltration, fewer denture dislodgments, and lower ratings of bothersomeness, corroborate those studies investigating the benefits of denture adhesive in preventing food infiltration.

## 1 INTRODUCTION

Despite the significant improvements dentures offer edentulous patients in terms of esthetics and function, some people remain unsatisfied with their dentures due to issues with prosthesis retention and stability (Alfadda, 2014). Denture adhesives can improve these factors by creating a seal along the inner, gum‐facing denture borders. This can help improve denture fit, increase retentive hold (Hoke et al., 2017; Papadiochou et al., 2015), and reduce denture movement while chewing, which can, as a result, help with mastication and reduce infiltration of potentially irritating food particles under the denture (Axe et al., 2018; Gosavi et al., 2013; Kumar et al., 2015; Ozcan et al., 2005; Papadiochou et al., 2015; Varghese et al., 2019).

Published methodologies for evaluating the performance of a denture adhesive at reducing food particle infiltration are limited in number. One such study had participants rate the perceived presence of food particles under their dentures with or without a denture adhesive after eating foods including celery, steak, and taffy/toffee apples. A significant difference was found in favor of the adhesive compared to no adhesive (Tarbet et al., 1980). Another study design described a quantitative methodology based on having participants chew a prescribed amount of peanuts while wearing their dentures. Peanut particles left under the intaglio surfaces were collected and evaluated and a significantly lower mass of peanuts was found when an adhesive was used compared to no adhesive use (Ahmad et al. 2009, 2010).

A previous study by some of the current authors (Atassi et al., 2019) utilizing the peanut chewing methodology (Ahmad et al., 2009, 2010) did not find a difference in food infiltration when a denture adhesive was compared to no adhesive. This was postulated to be due to noncontrolled amounts of denture adhesive being applied and to a nonoptimized method of peanut particle collection and handling following chewing. Here, a pilot method development study was carried out to ascertain if changes to the previous study methodology (Atassi et al., 2019) could be employed to better evaluate the mass of peanut particles that infiltrated under dentures when chewing, with an assessment of the pattern of denture adhesive application as the primary objective. This was followed by an efficacy study employing the refined methodology developed in the pilot with the primary objective of assessing the performance of a marketed denture adhesive compared to no adhesive. Peanuts were used in both studies as a representative, analyzable food; it is expected that similar results would be found with other foods that fragment into small particles during mastication.

In the efficacy study, a marketed denture adhesive (compared to no adhesive) was applied with a built‐in precision nozzle in a defined pattern to maximize the distribution of the adhesive across the denture, to minimize the excess application. Many denture adhesive tubes have wide nozzles with application instructions to apply bands or dabs of adhesive on the denture. The current nozzle is smaller and round, allowing more precise application of a thinner, targeted band of adhesive.

In the previous study (Atassi et al., 2019), participants were questioned regarding how their dentures felt during the chewing exercise. Here, in both the pilot and follow‐on efficacy study, secondary objectives included a comparison of the number of participant‐reported denture dislodgements while chewing and evaluation of participant ratings of the amount of peanut particles under their dentures and how bothered they were by them if present. These studies were carried out in participants with fair‐ or well‐fitting dentures only as the denture adhesive under evaluation is not intended as a substitute for poorly fitting dentures.

## 2 MATERIALS AND METHODS

Both the pilot and efficacy studies were single‐center, controlled, randomized, crossover trials conducted at a US‐based clinical research facility in full compliance with the International Council for Harmonisation of Technical Requirements for Registration of Pharmaceuticals for Human Use, all applicable local good clinical practice regulations and participant privacy requirements, and the ethical principles outlined in the Declaration of Helsinki. The pilot study (ClinicalTrials.gov: NCT03345108) was an open‐label, three‐treatment, three‐period study. The efficacy study (ClinicalTrials.gov: NCT03709810) was a single‐blind (technician weighing migrated peanut particles), two‐treatment, two‐period study. Final study protocols, informed consent forms, and other information that required preapproval were reviewed and approved by an independent institutional review board (US Investigational Review Board, Inc.: U.S.IRB2017SRI/12 and U.S.IRB2018SRl/05). Anonymized individual participant data and study documents can be requested for further research from www.clinicalstudydatarequest.com.

### 2.1 Participants

Unless otherwise stated, participant criteria were the same for both studies. Written, informed consent was provided by each participant before any study‐specific procedures. Eligible edentulous participants were aged between 18 and 85 years and in good physical and mental health. Participants had conventional, full, acrylic dentures in maxillary and mandibular arches, which they self‐reported getting food trapped under while eating. Dentures needed to be fair‐ to well‐fitting, defined as a Kapur Index (Olshan Modification) retention and stability index sum score ≥6 (Supporting Information: 1) for maxillary and mandibular dentures combined, with no individual stability or retention score <1 (Kapur, 1967; Olshan et al., 1992). Dentures were judged by the lead examiner as being well‐made and defined as having adequate vertical dimension, freeway space, horizontal occlusal relationships, and border extension with acceptable porosity, tissue surfaces, polished surfaces, color, and thickness. Additionally, each denture was required to have a peanut particle migration rating at screening >0 (see below). Denture‐bearing tissue scores were assessed (Kapur, 1967) (Supporting Information: 2), but the scores were not used for screening or stratification purposes.

Participants were ineligible if they were pregnant; breastfeeding; were receiving medication or had a medical condition that might interfere with the study; had any clinically significant or relevant oral abnormality; had an allergy/intolerance to study materials/ingredients or to peanuts/nuts or had received an investigational drug, cosmetic, or medical device within 30 days of screening. Participants could only have nonemergency dental/denture procedures performed during the study if allowed by the examiner.

### 2.2 Procedures

At the screening visits for both studies, participants underwent an oral soft tissue (OST) examination and were assessed for denture retention and stability. Participants were also assessed for adequate food migration under the dentures whereby they chewed and consumed a 30–32 g serving of peanuts in individual portions of approximately eight peanut halves at a time. Participants then rinsed their mouths with water for up to 10 s to help remove any peanut particles that had not migrated under the dentures. Visual assessment of the amount and degree of peanut particle migration under the dentures was conducted and graded accordingly by the examiner using a 4‐point scale from 0 (no peanut migration) to 3 (extensive peanut migration on the crest of the denture). To ensure only participants who experienced peanut particle infiltration were included in the study, participants with a score of 1–3 were randomized to the treatment sequence in which they would receive each condition according to a predetermined schedule generated by an independent statistical agency.

For the pilot study, a marketed denture adhesive (Super Poligrip® Free Denture Adhesive Cream containing polymethylvinyl ether/maleic acid; sodium–calcium mixed partial salt, petrolatum, cellulose gum, mineral oil; Haleon, Weybridge, UK; USA marketplace) was applied using a preloaded syringe at a dose of 1.0 g to the maxillary denture and 0.6 g to the mandibular denture when participants were randomized to the denture adhesive treatment arm. The treatment groups were as follows:

- Conventional pattern application: *Maxillary denture*: One central dab applied to the middle of the palatal region, and one dab each applied to the left and right premolar regions; *Mandibular denture*: One dab each applied to the left and right premolar regions.
- Continuous strips pattern application: *Maxillary denture:* Two short strips applied in the palatal region, and a single confluent strip applied to the middle of the alveolar ridge region, inside the buccolabial and posterior borders extending the entire arch; *Mandibular denture:* A confluent strip applied to the middle of the alveolar ridge region, extending the entire arch.
- No adhesive.

For the efficacy study, eligible participants were randomized (1:1) to one of two study groups in a crossover manner to first receive either:

- Test adhesive (COREGA Máximo Sellado/Selamento containing sodium–calcium mixed partial salt of polymethylvinyl ether/maleic acid, carboxymethylcellulose, petrolatum, mineral oil; Haleon) applied as detailed above in the continuous strips pattern via the precision nozzle incorporated into the primary packaging for a total dose of 1.0 g (±0.1 g) for the mandibular denture, and 0.6 g (±0.1 g) for the maxillary denture.
- No adhesive.

For both studies, the amount of adhesive applied to the denture was controlled by weighing the adhesive to be placed onto the denture. The amounts used (1.0 g to the maxillary and 0.6 g to the mandibular denture) were chosen to be representative of the amounts typically used in practice.

For both studies, during treatment visits, the use of any nicotine or nicotine‐containing products, or any other oral healthcare products other than the study products was prohibited while at the study site.

On test days, participants underwent an OST examination, and dentures were cleaned and dried before adhesive application/denture insertion; participants rinsed their mouth with water and expectorated before denture insertion. Adhesive/no adhesive was applied (per randomization schedule) and dentures were placed in the mouth; then the participant pressed the denture firmly in place and bit down for a few seconds to secure the hold. At 60 (±5) min after denture insertion, each participant was given a standardized portion of peanuts (30–32 g) to consume following a prescribed chewing and swallowing method whereby participants sequentially chewed approximately eight peanut halves for at least 20 s before swallowing. Participants were allowed small sips of water during peanut consumption to aid chewing and swallowing. They were provided with a tick‐box sheet and instructed to record each incident of denture dislodgement (of either denture) that occurred while chewing the peanuts.

After peanut consumption, participants gently rinsed their mouths with water for 5 s in the pilot study. This rinsing step was increased to 10 s for the follow‐on efficacy study as it was discovered that 5 s was not sufficient to remove all residual peanut particles from around the mouth that had not been retained under the dentures. The examiner removed any peanut particles from the vestibular and sublingual areas (maxillary and mandibular), then carefully extracted the mandibular denture. They then removed and collected any peanut particles and residual adhesive, first from the lower edentulous ridge using gauze, then the maxillary denture. Respective gauzes for each denture were retained in separate, marked beakers.

Any residual peanut particles present on the denture surface other than those on the intaglio surface were discarded. In each study, with approximately five participants per group, the denture intaglio surface was photographed such that a variety of denture sizes and fits were represented. These images were for visualization purposes only and were not analyzed.

Maxillary and mandibular dentures, together with their corresponding gauze, were placed in separate beakers of warm deionized (DI) water and sonicated for at least 30 min to loosen adhering peanut particles. Any peanut pieces remaining on the prosthesis or gauze were then rinsed out into the beaker with fresh DI water. The gauze pieces were discarded and the dentures were cleaned and returned to the participant. The contents of the beaker (water, adhesive, saliva, and peanut particles) were heated to boiling with frequent stirring to facilitate the dissolution of any remaining adhesive and then strained through a standard #60 testing sieve. The remaining peanut particles were repeatedly washed with hot DI water to remove residual saliva and contaminants, air‐dried overnight, then oven dried for 5 h at 40°C. Once cooled, the mass (mg) of the dry peanut particles that had migrated under each denture was determined.

In both studies, following food infiltration testing and denture removal on test days, participants completed a questionnaire on their experience during chewing and whether they had been aware of peanut particles under their dentures (Yes/No answer). Participants who answered “yes” completed additional questions relating to their subjective assessment of the amount of peanut particles under the dentures (1 = none to 10 = numerous), and how bothered they were by these (1 = not at all bothered to 10 = extremely bothered). Additionally, in the pilot study, participants rated how irritating the peanut particles were (1 = not at all irritating to 10 = extremely irritating).

Safety was assessed by OST examination at screening and before/after each assessment at the treatment visits, through a collection of adverse events (AEs) and medical device incidents. Abnormalities and AEs reported after the participant's first use of treatment were considered treatment‐emergent adverse events (TEAEs).

### 2.3 Efficacy measurements

For both studies, the primary efficacy evaluation was a combined mass of peanuts under both dentures when denture adhesive was applied per the conventional pattern. For the efficacy study, secondary efficacy endpoints included the mass of peanut particles recovered separately under each denture. Additional secondary endpoints were the number of participant‐reported denture dislodgements and participant responses to the postpeanut chewing questionnaire. For the pilot study, exploratory efficacy evaluations included these plus a combined mass of peanuts under both dentures when the denture adhesive was applied per continuous strip pattern or when no adhesive was used and the mass of peanuts under combined dentures in participants with a low or high Kapur–Olshan (KO) score with each treatment.

### 2.4 Statistical analysis

For the pilot study, as the purpose was not to compare treatment arms but to understand if the food occlusion model was applicable to different patterns of adhesive application, the study was not blinded and no formal statistical analysis was performed. Approximately 48 (maximum 50) participants were planned to be enrolled. Recruitment was targeted to achieve 50 ± 10% of participants with low (score of 6–14, clinically fair to good denture retention and stability) or high (score of 15–18, clinically very good denture retention and stability) KO Index sum scores.

In the efficacy study, it was planned to screen 53 participants and randomize 52 to ensure that 48 evaluable participants would complete the study. It was estimated that this sample size would provide 91% power to detect a multiplicative difference of 2.25 (0.3522 on the logarithmic scale) between the Test adhesive and no adhesive in the mean mass of peanut particles recovered from maxillary and mandibular dentures (based on the pilot study results). These were calculated using power analysis and sample size (PASS) software (NCSS Statistical Software, 2019) for a two‐period crossover trial and using the Sw option for the variability estimate as 0.5075. An extensive simulation conducted in the PASS study supported the above calculations.

Primary and secondary efficacy analyses for both studies were based on a modified intent‐to‐treat (mITT) population, including all participants who were randomized, received at least one dose of study treatment, and had at least one assessment of efficacy. For the efficacy study, primary and secondary analysis of food infiltration was performed using a mixed model analysis of variance (ANOVA) with Log_10_ peanut mass as a response, study treatment and period as fixed explanatory effects, and participant as a random effect. The geometric mean (GM), geometric coefficient of variation (CV), and 95% confidence interval (CI) were presented. All statistical tests of the hypothesis were two‐sided and employed a level of significance of *α* = .05. The number of denture dislodgements according to the number of participants was presented as a frequency distribution. Analysis of denture dislodgements (mITT population) was performed by nonparametric testing, applied to obtain inferential statistics, as assumptions underlying the ANOVA model could not be supported. Analysis was performed using the Wilcoxon signed‐rank test and the Hodges–Lehmann method.

For participants’ responses to questionnaires, descriptive statistics, mean score, and standard error (SE) were calculated for each question by the treatment group and frequency graphs were constructed. Analysis of participants’ responses to questions was performed using the methods described for the number of denture dislodgements.

## 3 RESULTS

**Figure 1:**
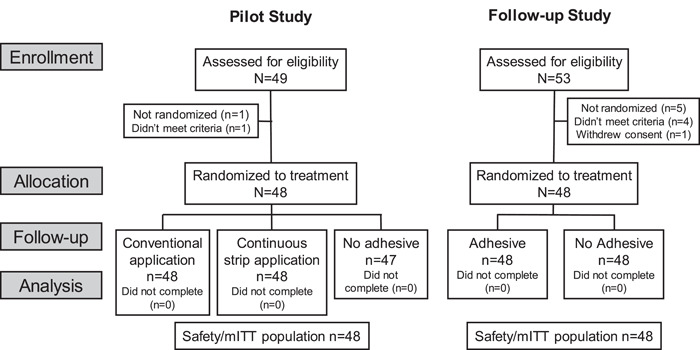
Study flow schematic. mITT, modified intent‐to‐treat.

In the pilot study, the first participant was enrolled in November 2017; the final participant completed the study in December 2017. Of the 49 participants screened, 48 were randomized to a treatment sequence and were included in the mITT and safety populations (Figure 1). Most participants were females (70.8%) and were either of White/European heritage (91.7%%) or African American/African heritage (8.3%). The mean age was 65.0 years (standard deviation [SD] 12.60: range 31–81 years).

For the efficacy study, a total of 53 participants were screened for eligibility and 48 were randomized and completed treatment (Figure 3). Most participants were females (*n* = 33; 68.8%) and were either White/European heritage (*n* = 43; 89.6%) or African American/African heritage (*n* = 4; 8.3%). The mean age was 65.1 years (SD 10.77: range 31–81 years).

### 3.1 Denture‐related characteristics

In the pilot study, current denture history was comparable for maxillary and mandibular dentures, with mean denture ages of 11.4 years (SD 12.31 years; range: 1.0–52.0 years) and 12.0 years (SD 12.25 years; range: 1.0–52.0 years), respectively. Fewer (25%) participants with high KO scores at screening were recruited than planned, with 75% having a low KO score; however, sufficient participant numbers were recruited in each subgroup (high or low KO scores) to allow for adequate analysis.

In the efficacy study, participants had an average duration of denture use of 23.0 years (SD 19.14 years; range 0.1–63.0 years) for maxillary dentures, and 22.8 years (SD 18.83 years; range 0.1–63.0 years) for mandibular dentures. Current denture history was comparable for maxillary and mandibular dentures, with mean denture ages of 12.3 years (SD 13.57 years; range: 0.1–54.0 years) and 12.2 years (SD 13.54 years; range: 0.1–54.0 years), respectively. No participants had dentures with “poor retention and stability” (<6 Kapur Index total score); 1 (2.1%) had “fair retention and stability” (6–9 total score); 24 (50.0%) had “good retention and stability” (10–14 total score), and 23 (47.9%) had “very good retention and stability” (>14). The mean combined score of 14.8 for the fit assessment (Kapur Index) was indicative of overall very good denture fit and stability at baseline. Denture adhesive was currently being used at screening on maxillary dentures by 17 participants (35.4%) and on mandibular dentures by 20 participants (41.7%). Denture‐bearing tissue scores can be found in Supporting Information: Table 1; briefly, the majority of participants had resilient to firm tissue resiliency, and low to medium border tissue attachment.

### 3.2 Peanut particle mass

**Figure 2:**
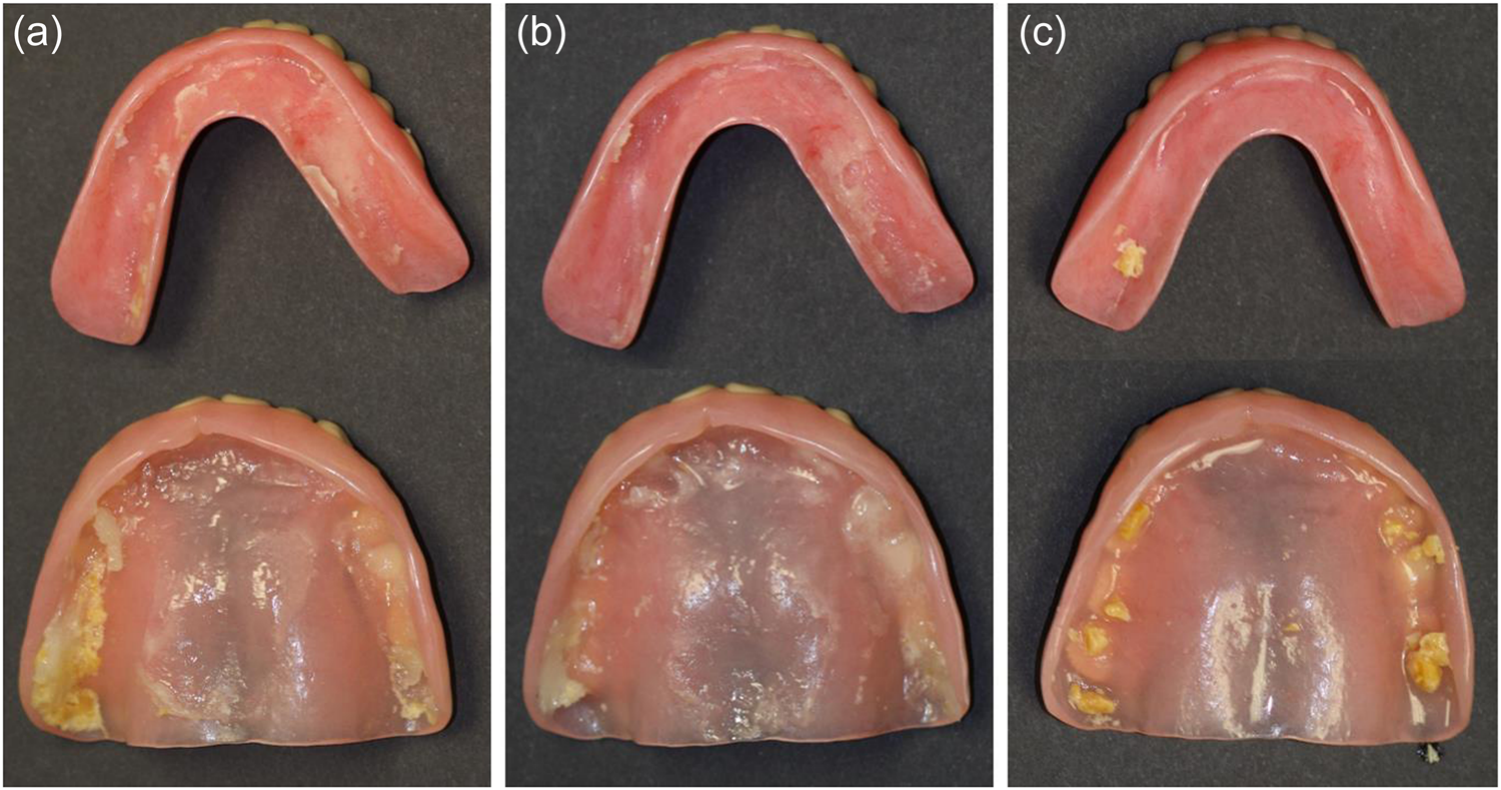
Representative photographs from the pilot study of dentures before collection of peanut particles and residual denture adhesive (when applied) per (a) conventional application, (b) continuous application, and (c) no adhesive. Photographs adjusted for contrast and brightness. Black arrows, denture adhesive; red arrows, peanut particles.

Figure 2 shows typical photographs of dentures after the chewing challenge with and without adhesive use, demonstrating both residual denture adhesive and peanut particles. It can be deduced from these photos that peanut particles have ingressed under the dentures, especially where no adhesive was applied, with particles particularly present in the areas of the dentures contacting the alveolar ridge in the case of maxillary dentures.

Graphs and tables for the pilot study are held in the section of Supporting Information. For the pilot study (Supporting Information: Table 2 and Figure 1), peanut particle mass data were positively skewed with mean masses being considerably greater than medians; therefore, both median and mean masses are discussed.

Without adhesive, a greater median (11.0 mg, range 0.1–700.0 mg) and mean (58.4 mg, SE 20.48 mg) mass of peanut particles migrated under the mandibular dentures than the maxillary ones (median 7.7 mg, range 0.1–213.4 mg; mean 20.1 mg, SE 5.79 mg). This was the same when the adhesive was applied by the conventional pattern (mandibular median 3.4 mg, range 0.1–27.1 mg; mean 5.8 mg, SE 0.94 mg; maxillary median 2.7 mg, range 0.1–89.2 mg; mean 6.0 mg, SE 1.95). However, when applied by the continuous pattern, there was a slightly lower median and mean for the mandibular dentures (median 1.4 mg, range 0.0–20.0 mg; mean 3.8 mg, SE 0.72 mg) compared with the maxillary ones (median 1.8 mg, range 0.0–57.3 mg; mean 5.6 mg, SE 1.41 mg). When assessed by low or high KO scores, there were few differences for the conventional application or no adhesive; however, for the continuous pattern group, the median mass of peanuts under the combined dentures was higher in those with a low KO score (median 5.8, range 0.2–75.6 mg; mean 10.7, SE 2.35) compared with those with a high KO score (median 2.1, range 0.2–36.7 mg; mean 5.7, SE 2.94) (Supporting Information: Table 2)

For the efficacy study, the combined GM mass of peanut particles recovered from both maxillary and mandibular dentures was lower when the Test adhesive was used (5.56 mg; CV 1.160 mg) compared to no adhesive (29.13 mg; CV 1.160 mg) (Table 1). The primary analysis of the between‐group GM ratio deduced as Test adhesive over no adhesive, demonstrated a statistically significant difference in favor of the Test adhesive (0.19 [95% CI: 0.12, 0.30]; *p* < .0001) (Table 1). The GM equates to 5.2 times lower amount of peanut particle infiltration with the GM ratio indicating that the mass of peanut particles under the denture when using the Test adhesive is 19% of that when using no adhesive. Similarly, the GM mass of peanut particles recovered from the maxillary (5.9 times lower) or mandibular (4.8 times lower) dentures separately was statistically significantly lower with the Test adhesive, with, respectively, 17% and 21% of the infiltration detected with no adhesive (Table 1).

**Table 1:** Efficacy (follow‐on) study the mass of peanut particles (mg), the geometric mean ratio between treatment (modified intent‐to‐treat cohort)

Mass (mg)	Adhesive	No adhesive
Combined		
Mean (SE) [min–max]	17.16 (7.475) [0.2–346.0]	78.55 (20.695) [1.3–860.0]
GM (CV) [95% CI]	5.56 (1.160) [3.72, 8.31]	29.13 (1.160) [19.48, 43.56]
GM ratio [95% CI], *p* value	0.19 [0.12, 0.30], *p* < .0001	
Maxillary		
Mean (SE) [min–max]	4.85 (2.233) [0.0–106.8]	22.02 (6.835) [0.6–264.7]
GM (CV) [95% CI]	1.39 (1.216) [0.92, 2.11]	8.17 (1.216) [5.39, 12.38]
GM ratio [95% CI], *p* value	0.17 [0.11, 0.27], *p* < .0001	
Mandibular		
Mean (SE) [min–max]	12.31 (7.141) [0.0–345.2]	56.53 (19.129) [0.0–855.5]
GM (CV) [95% CI]	2.75 (1.795) [1.63, 4.64]	13.07 (1.795) [7.75, 22.02]
GM ratio [95% CI], *p* value	0.21 [0.10, 0.43], *p* < .0001	

*Note*: Analysis was performed using the ANOVA model with Log10 peanuts mass as response variable; study product and period as fixed explanatory effects and participant as a random effect; a GM ratio of <1 implies the mass of peanuts under the denture when using the Test adhesive is reduced to that percentage (e.g., 19% for Combined) of no adhesive.

Abbreviations: ANOVA, analysis of variance; CI, confidence interval; CV, geometric coefficient of variation; GM, geometric mean, adhesive over no adhesive; SE, standard error.

### 3.3 Denture dislodgements

Pilot study data are presented in Supporting Information: Table 3. Briefly, the mean number of denture dislodgements was 0.35 (SE 0.113) for the conventional application group; 0.25 (SE 0.138) for the continuous application group, and 1.64 (SE 0.255) for the no adhesive group.

**Figure 3:**
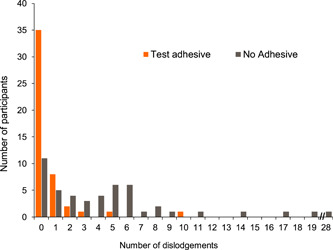
Efficacy (follow‐on) study frequency graph of participant‐rated number of denture dislodgements by treatment (modified intent‐to‐treat population)

For the efficacy study, the number of denture dislodgments experienced by participants while chewing is shown in Figure 3. During the chewing procedure, a greater number of participants reported no denture dislodgments when using the Test adhesive (*n* = 35; 72.9%) compared to without adhesive (*n* = 11; 22.9%). A mean of 0.6 (SE 0.24) dislodgements were reported when using the Test adhesive compared to a mean of 4.7 (SE 0.80) without adhesive. A lower median number of patient‐reported denture dislodgements was evident with the Test adhesive compared to no adhesive (0.0 vs. 4.0, respectively). The median difference (Test adhesive minus no adhesive) following analysis using the Wilcoxon signed‐rank test was −3.00 and was significantly different in favor of the Test adhesive (*p* < .001). The point estimate (Hodges–Lehmann estimates of median differences) was −3.00 (95% CI −4.50, −2.00).

### 3.4 Participant questionnaires

Pilot study data are presented in Supporting Information: Table 4. Briefly, 6.3% (*n* = 3) of conventional application, 6.3% (*n* = 3) of continuous application, and 70.8% (*n* = 34) of no adhesive participants reported being aware of peanuts under dentures.

**Figure 4:**
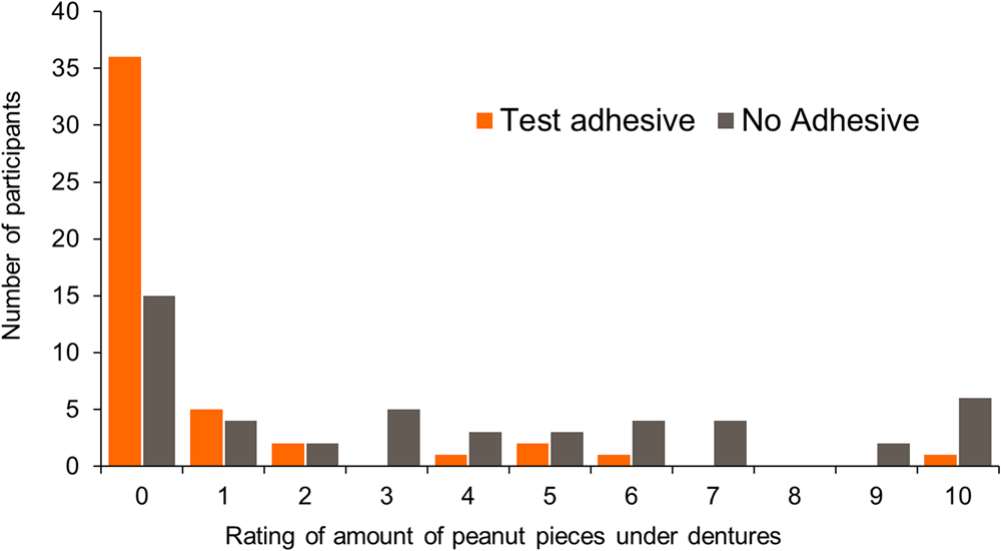
Efficacy (follow‐on) frequency graph of participant ratings of level of awareness of peanut particles under dentures by treatment (modified intent‐to‐treat population)

For the efficacy study, overall, 12 participants (25.0%) using the Test adhesive and 33 (68.8%) without adhesive reported being aware of peanut particles under their dentures and answered two further questions. When using the Test adhesive, participants reported a mean score of 0.8 (SE 0.29) on the “amount of peanut pieces under denture” rating scale (0 = none to 10 = lots); no adhesive results showed a mean score of 3.8 (SE 0.52). The median between‐group difference was in favor of the Test adhesive versus no adhesive and was statistically significant (−1.50; point estimate −2.50; 95% CI: −4.50, −1.50; *p* < .0001). (Figure 4)

**Figure 5:**
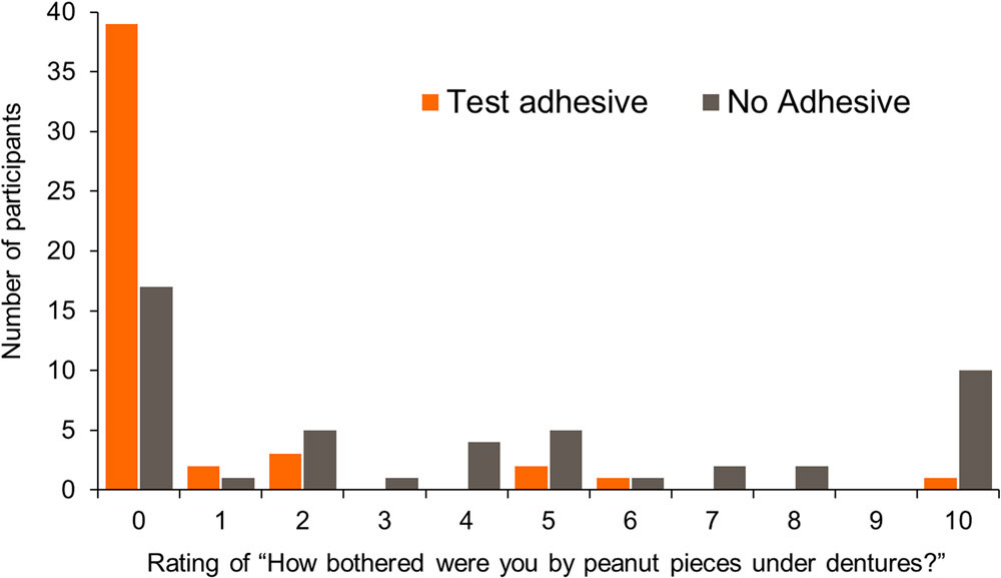
Efficacy (follow‐on) frequency graph of participant ratings of level of how bothersome peanut pieces felt under denture by treatment group (modified intent‐to‐treat population)

When using the Test adhesive, participants reported peanut pieces under their dentures to be less bothersome (mean score 0.7, SE 0.28) than during the no adhesive period (mean score 4.0, SE 0.57) (Figure 5). The median between‐group difference in favor of Test adhesive use versus no adhesive was also statistically significant (−2.00; point estimate −3.00; 95% CI: −4.50, −1.50; *p* < .0001).

### 3.5 Safety

For the pilot study, there were no TEAEs, serious AEs, medical device incidents, or deaths reported during the study. For the efficacy study, overall, six TEAEs were reported, all classified as oral: hyperkeratosis (*n* = 1 for Test adhesive; *n* = 2 for no adhesive); tongue biting (*n* = 1 for Test adhesive); mouth injury (*n* = 1 for no adhesive), and a traumatic ulcer (*n* = 1 for no adhesive). There were no treatment‐related TEAEs, serious TEAEs, deaths during the study, or TEAEs leading to premature study treatment discontinuation.

## 4 DISCUSSION

The accumulation of food under dentures can be a considerable issue for wearers, even when dentures have been fitted appropriately (Brunello & Mandikos, 1998; Gosavi et al., 2013). This was highlighted here in individuals with well‐fitting dentures (mostly rated as “good” or “very good” retention and stability scores), where around 70% of participants in both studies reported that they were aware of peanuts under their dentures when denture adhesive was not used.

Both studies examined the efficacy of a measured amount of a marketed denture adhesive applied in thin strips with a precision nozzle compared to no adhesive with regard to food infiltration under dentures. This is in contrast to traditional application nozzles, where the denture adhesive is intended to be applied more coarsely as dabs on the denture intaglio surface (the pilot study “conventional application” pattern). In the pilot study, the pattern of adhesive application had only a small effect; however, the continuous strip application resulted in a numerically lower median/mean mass of peanuts recovered from under the dentures compared to the conventional application. It should be noted that in practice, the amount of denture adhesive required to ensure adequate denture hold and to prevent food particle ingress may be more or less than the amount dosed in this study owing to differences in user's denture fit, size of a denture, quality of underlying soft tissue and user preference. Both studies allowed a period of 60 min between the fitting of the denture and the commencement of testing. This was to ensure the adhesive had fully hydrated before testing, allowing the denture hold to stabilize.

In the follow‐on efficacy study, the primary objective was met with a greater than fivefold reduction in peanut infiltration under dentures when using the Test adhesive compared to no adhesive use. The denture adhesive was shown to be effective in reducing the infiltration of peanut particles under both maxillary and mandibular dentures when analyzed separately or together. A higher mass of peanut particles was observed under the mandibular dentures compared to the maxillary ones in both treatment groups. This was also found in the pilot study and previous studies (Ahmad et al., 2010; Munoz et al., 2012) and is postulated to be due to poorer retention of the mandibular denture based on the activity and movements of the mandible, tongue, and facial musculature during mastication (Bohnenkamp & Garcia, 2007). The pattern of food distribution around the mouth while chewing and the effects of gravity may also result in more peanut particles gathered in the mandibular denture region during chewing. However, while the mass of peanuts under the mandibular dentures was greater, the GM ratio was similar for both dentures as well as the combined dentures, demonstrating that the adhesive works equally well with maxillary and mandibular dentures at preventing food infiltration.

Another finding of the efficacy study was that only 27.1% of those using the Test adhesive reported any denture dislodgments during the chewing procedure used, compared to 77.1% of those when not using adhesive. More participants in the no adhesive groups also reported multiple (>1) dislodgements. This is in agreement with previous studies examining the maximum incisal bite force a person can exert until denture dislodgement, which generally shows that a greater bite force is achievable when denture adhesive is used compared to no adhesive use (Axe et al., 2018; Jose et al., 2018; Varghese et al., 2019). Fewer denture dislodgements with the use of denture adhesive may be related to reduced movement of the denture while chewing (Tarbet et al., 1980) and supports the reduction in peanut particle infiltration observed in the primary analysis (Munoz et al., 2012). The higher infiltration of peanuts under mandibular dentures may be related to a greater number of mandibular denture dislodgements, as has been shown previously (Grasso et al., 2000); however, participants in this study were not asked to report which denture had been dislodged as it was considered too onerous for the participants to be able to accurately report dislodgements separately while chewing peanuts in the prescribed fashion. This could be examined in further studies.

Previous denture adhesive studies have also seen similar improvements in participant ratings for denture confidence, comfort, satisfaction, and movement when denture adhesive was used compared to no adhesive (Atassi et al., 2019; Munoz et al., 2012). In this study, participants were more specifically asked to rate the amount of peanut particles they perceived as being under their dentures. It was shown that significantly fewer participants were aware of peanuts under their dentures while using the Test adhesive (25.0%) compared to no adhesive (68.8%). Scores for the question posed to those who perceived peanut particles under their dentures—“how bothered were you by the peanut pieces under your denture?”—were also significantly lower with the Test adhesive. These results show that participants’ perceived efficacy of the marketed denture adhesive was significantly higher compared to no adhesive and reflects the objective measurements shown when peanut particles were weighed.

A previous food infiltration study by a number of the current authors also investigated the use of an adhesive compared to nonadhesive use but did not find a significant difference between them (Atassi et al., 2019). This was postulated to be due to several factors including controlling the amount of adhesive applied to each denture and having a more precise method of collection and processing of the peanut particles. As such, the methodology to apply denture adhesive and collect peanut particles was refined in the pilot study described here. These improvements in the methodology provided results in the efficacy study where statistically significant differences were shown, suggesting that this refined protocol is suitable for examining the effects of treatments on food infiltration under dentures.

One potential consideration in these studies was that there was a large range of denture age and denture‐wearing experience. However, as this was a crossover study, there was no difference between the groups regarding this. As dentures were judged on their Kapur Index for retention and stability as well as the visual examination of fit and condition, the age of the denture should not affect the results. Test adhesive use was generally well tolerated with no treatment‐related TEAEs.

In conclusion, the use of denture adhesive, applied in a specified amount and pattern with a precision application nozzle, was found to result in a lower mass of peanut particles found under dentures and also improve participant perception of such. This could be related to both better denture adherence to the gum as well as to the finding of fewer denture dislodgements while chewing. These findings add further weight to the utility of denture adhesive in those with well‐fitting and well‐made full maxillary or mandibular dentures. As complaints of food infiltration are common amongst denture wearers, reduction of such could lead to higher enjoyment of food and a greater range of food choices.

## AUTHOR CONTRIBUTIONS

All authors contributed to the design and reporting of the study, and/or were involved in its conduct. All authors had access to the final study report, made contributions to the development of the manuscript, had final responsibility for the decision to submit, and approved the manuscript.

## CONFLICTS OF INTEREST

R. V. and G. R. B. are employees of the study sponsor GSK Consumer Healthcare, now known as Haleon. M. A. was an employee of GSK Consumer Healthcare at the time of the research. K. M. and J. M. are Directors of Salus Research, which has received funding from Haleon.

## Supporting information

Supporting information.

## Data Availability

Anonymized individual participant data and study documents can be requested for further research from www.clinicalstudydatarequest.com
