# Supplementary material for: A randomized clinical study to assess the performance of a marketed denture adhesive in a model of food infiltration in healthy, edentulous adults
Source: Clin Exp Dent Res. 2022 Dec 13;9(2):388–97. doi: 10.1002/cre2.703 (PMC10098280; doi:10.1002/cre2.703)
Supplement: Supplementary file 1 — Supporting information. [file CRE2-9-388-s001.docx]

**Supplemental data**

**Supplemental Information 1: Well-fit assessment, Kapur (Olshan modification) Index**

Each denture (upper and lower) was examined for retention and stability using the Kapur

Index (Olshan Modification)^1,2^ by an examiner with expert knowledge of prosthodontics. A sum score (upper + lower) of ≥ 6 was required for inclusion.

**Retention:** With gloved hands, the examiner attempted to unseat the upper and lower denture by applying an opposing vertical force at the canine/lateral incisor region of the denture. The examiner scored retention using the following criteria:

- 5 = Excellent: Denture offers excellent resistance to vertical pull and lateral force
- 4 = Very Good: Denture offers very good resistance to vertical pull and lateral force
- 3 = Good: Denture offers moderate resistance to vertical pull and lateral force
- 2 = Fair: Denture offers moderate resistance to vertical pull and little or no resistance to lateral forces
- 1 = Poor: Denture offers slight resistance to vertical pull and little or no resistance to lateral force
- 0 = No retention: When the denture is seated in place, it displaces itself

**Stability:**

With gloved hands, the examiner attempted to rock the seated dentures by placing alternate horizontal force at the cuspid and contralateral molar regions of the upper and lower dentures. The examiner scored denture stability as 0 - 4 using the following criteria:

- 4 = Excellent: When denture base offers no rocking on its supporting structures under pressure
- 3 = Good: When denture base has very slight rocking on its supporting structures under
- pressure
- 2 = Fair: When denture base has slight rocking on its supporting structures under pressure
- 1 = Poor: When denture base has moderate rocking on its supporting structures under pressure
- 0 = No stability: When denture base has extreme rocking under pressure

1. Olshan AM, Ross NM, Mankodi S, Melita S. A modified Kapur scale for evaluating denture retention and stability: methodology study. American Journal of Dentistry 1992;5(2):88–90.

2. Kapur KK. A clinical evaluation of denture adhesives. Journal of Prosthetic Dentistry 1967;18(6):550-8.

**Supplemental Information 2: Denture Bearing Tissue Score**

The denture bearing tissue score was assessed by an examiner with expert knowledge of prosthodontics and recorded for the maxillary and mandibular dentures.^1^ There were no eligibility requirements associated with this measure in this clinical trial.

- Ridge shape (for both maxillary and mandibular): 1 = Flat; 2 = V-shaped; 3 = Shaped between U and V; 4 = U-shaped
- Tissue resiliency (for both maxillary and mandibular): 1 = Flabby; 2 = Resilient; 3 = Firm
- Location of border tissue attachment
  - Maxillary arch: 1 = Low; 2 = Medium; 3 = High
  - Mandibular arch: 1 = High; 2 = Medium;* 3 = Low*

Note: Due to an inconsistency observed in the original printed publication, the two descriptors above marked by an asterisk (*) had been modified (by inverting their order) to better reflect the authors’ intent and align with the grading scale.

1. Kapur KK. A clinical evaluation of denture adhesives. Journal of Prosthetic Dentistry 1967;18(6):550-8.

**Supplemental Table 1: Denture bearing tissue scores (follow-on study)**

|  |  | **Maxillary dentures** | **Mandibular dentures** |
| --- | --- | --- | --- |
| **Ridge shape** | Flat | 0 | 7 (14.6) |
|  | V-shaped | 1 (2.1) | 15 (31.3) |
|  | Between U and V | 0 | 4 (8.3) |
|  | U-shaped | 47 (97.9) | 22 (45.8) |
| **Tissue resiliency** | Flabby | 1 (2.1) | 1 (2.1) |
|  | Resilient | 3 (6.3) | 14 (29.2) |
|  | Firm | 44 (91.7) | 33 (68.8) |
| **Border tissue attachment** | Low | 40 (83.3) | 17 (35.4) |
|  | Medium | 7 (14.6) | 18 (37.5) |
|  | High | 1 (2.1) | 12 (27.1) |

**Supplemental Table 2. Mass of peanuts (mg) under dentures (modified intent-to-treat population) from pilot study**

| **Dentures** | **Conventional application (n=48)** | | **Continuous application (n=48)** | | | **No adhesive**  **(n=47)** | |
| --- | --- | --- | --- | --- | --- | --- | --- |
|  | **Mean (SE)** | **Median (range)** | | **Mean (SE)** | **Median (range)** | **Mean (SE)** | **Median (range)** |
| **Combined** | 11.8 (2.34) | **6.5** (1.0–103.5) | | 9.4 (1.92) | **5.0** (0.2–75.6) | 78.5 (23.13) | **23.9** (2.3–863.8) |
| Low KO score | (n = 36) | | (n = 36) | | | (n = 35) | |
|  | 10.8 (1.63) | **6.3** (1.3–38.5) | | 10.7 (2.35) | **5.8** (0.2–75.6) | 94.9 (30.57) | **24.3** (2.3–863.8) |
| High KO score | (n = 12) | | (n = 12) | | | (n = 12) | |
|  | 15.0 (8.21) | **6.8** (1.0–103.5) | | 5.7 (2.94) | **2.1** (0.2–36.7) | 30.8 (7.48) | **23.2** (5.5–97.7) |
| **Mandibular** | 5.8 (0.94) | **3.4** (0.1–27.1) | | 3.8 (0.72) | **1.4** (0.0–20.0) | 58.4 (20.48) | **11.0** (0.1–700.9) |
| **Maxillary** | 6.0 (1.95) | **2.7** (0.1–89.2) | | 5.6 (1.41) | **1.8** (0.0–57.3) | 20.1 (5.70) | **7.7** (0.1–213.4) |

KO: Kapur-Olshan; SE: standard error

**Supplemental Table 3. Participant-reported denture dislodgements during chewing (modified intent-to-treat population) from pilot study**

| **Denture dislodgements** | **Conventional application (n=48)** | **Continuous application (n=48)** | **No adhesive (n=48)** |
| --- | --- | --- | --- |
| Mean number of denture dislodgements, n (SE) | 0.25 (0.113) | 0.25 (0.138) | 1 .64 (0.255) |
| Denture dislodgements, n (%) |  |  |  |
| 0 | 42 (87.5) | 44 (91.7) | 17 (35.4) |
| 1 | 3 (6.3) | 1 (2.1) | 7 (14.6) |
| 2 | 1 (2.1) | 1 (2.1) | 12 (25.0) |
| 3 | 1 (2.1) | 0 | 5 (10.4) |
| 4 | 1 (2.1) | 1 (2.1) | 2 (4.2) |
| 5 | 0 | 1 (2.1) | 2 (4.2) |
| 6 | 0 | 0 | 1 (2.1) |
| 7 | 0 | 0 | 1 (2.1) |
| Missing | 0 | 0 | 1 |

**Supplemental Table 4. Participant questionnaire responses (modified intent-to-treat population) in pilot study**

|  | **Conventional application (n=48)** | **Continuous application (n=48)** | **No adhesive (n=48)** |
| --- | --- | --- | --- |
| **Participants aware of peanuts under dentures, n (%)** | 3 (6.3%) | 3 (6.3%) | 34 (70.8%) |
| **Questions in participants aware of peanuts**^†^ | (n = 3) | (n = 3) | (n = 34) |
| Amount of peanuts perceived under dentures^‡^, mean (SE) | 3.3 (0.88) | 3.7 (0.67) | 3.3 (0.21) |
| Irritation of peanuts under dentures^§^, mean (SE) | 3.0 (1.00) | 4.7 (2.19) | 3.9 (0.39) |
| Bothered by peanuts under dentures^¶^, mean (SE) | 3.3 (1.45) | 5.0 (2.52) | 3.8 (0.30) |

^†^Only participants who answered that they were aware of peanut particles under their dentures completed additional questions

^‡^Scored on a scale of 1 (none) to 10 (numerous)

^§^Scored on a scale of 1 (not at all irritating) to 10 (extremely irritating)

^¶^Scored on a scale of 1 (not at all bothered) to 10 (extremely bothered)

**Supplemental Figure 1:** **Mass of peanuts under dentures according to treatment group (modified intent-to-treat population) from pilot study; N=48**

**
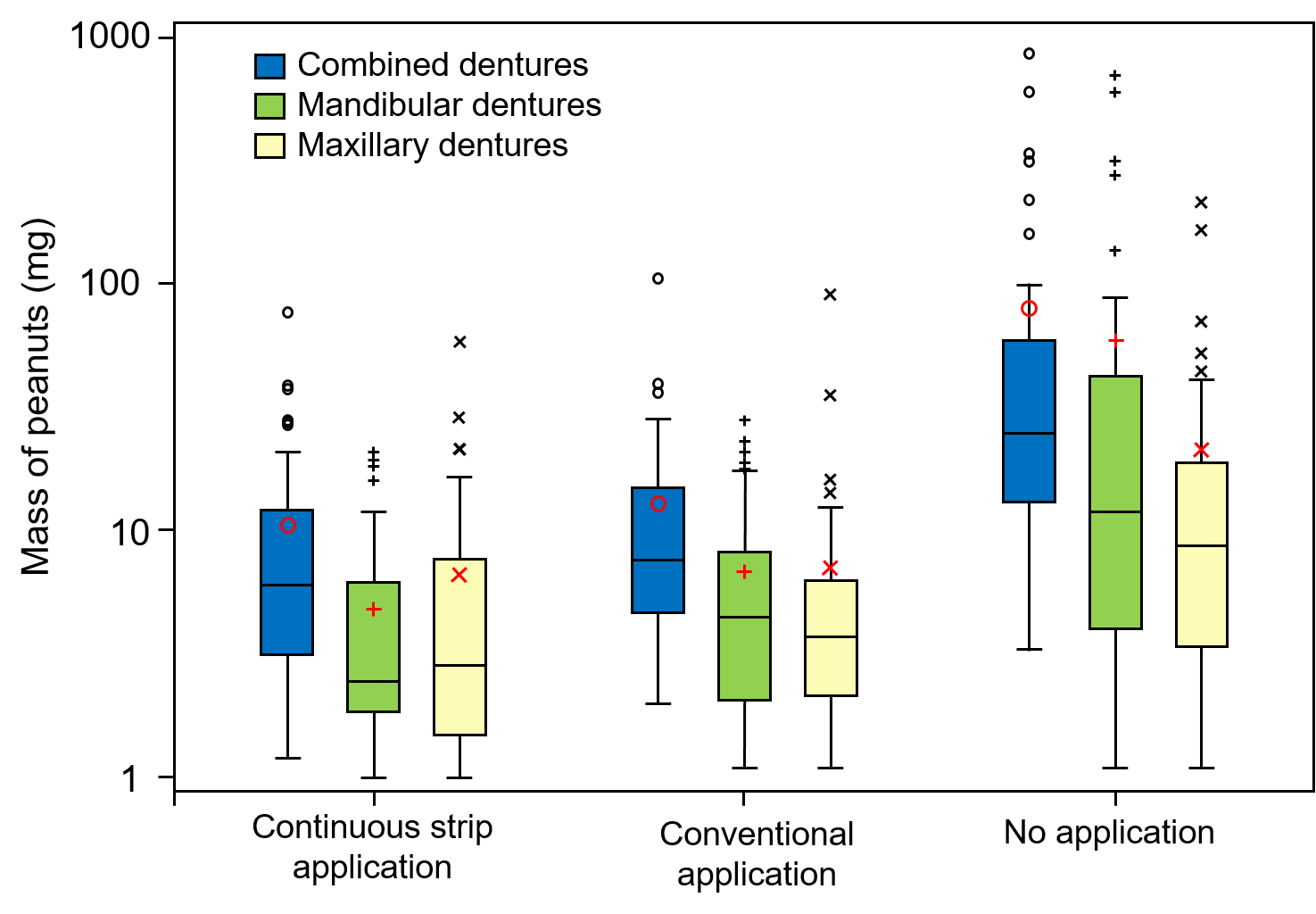
**

Mass of peanuts (mg) displayed on a logarithmic log(x+1) axis; data are not log transformed.

Group mean is displayed as larger red circles (combined dentures), crosses (mandibular dentures) or diagonal crosses (maxillary dentures); the horizontal line in the box represents the group median, outliers are represented as smaller black circles (combined dentures), crosses (mandibular dentures) or diagonal crosses (maxillary dentures); the box represents the interquartile range and the whiskers represent the non-outliers minimal and maximum values.
